# Pre-Transplant Donor-Specific T-Cell Alloreactivity Is Strongly Associated with Early Acute Cellular Rejection in Kidney Transplant Recipients Not Receiving T-Cell Depleting Induction Therapy

**DOI:** 10.1371/journal.pone.0117618

**Published:** 2015-02-17

**Authors:** Elena Crespo, Marc Lucia, Josep M. Cruzado, Sergio Luque, Edoardo Melilli, Anna Manonelles, Nuria Lloberas, Joan Torras, Josep M. Grinyó, Oriol Bestard

**Affiliations:** 1 Experimental Nephrology Laboratory, Bellvitge Biomedical Research Institute (IDIBELL), Barcelona, Spain; 2 Renal Transplant Unit, Nephrology department, Bellvitge University Hospital, Barcelona, Spain; INSERM, FRANCE

## Abstract

Preformed T-cell immune-sensitization should most likely impact allograft outcome during the initial period after kidney transplantation, since donor-specific memory T-cells may rapidly recognize alloantigens and activate the effector immune response, which leads to allograft rejection. However, the precise time-frame in which acute rejection is fundamentally triggered by preformed donor-specific memory T cells rather than by *de novo* activated naïve T cells is still to be established. Here, preformed donor-specific alloreactive T-cell responses were evaluated using the IFN-γ ELISPOT assay in a large consecutive cohort of kidney transplant patients (n = 90), to assess the main clinical variables associated with cellular sensitization and its predominant time-frame impact on allograft outcome, and was further validated in an independent new set of kidney transplant recipients (n = 67). We found that most highly T-cell sensitized patients were elderly patients with particularly poor HLA class-I matching, without any clinically recognizable sensitizing events. While one-year incidence of all types of biopsy-proven acute rejection did not differ between T-cell alloreactive and non-alloreactive patients, Receiver Operating Characteristic curve analysis indicated the first two months after transplantation as the highest risk time period for acute cellular rejection associated with baseline T-cell sensitization. This effect was particularly evident in young and highly alloreactive individuals that did not receive T-cell depletion immunosuppression. Multivariate analysis confirmed preformed T-cell sensitization as an independent predictor of early acute cellular rejection. In summary, monitoring anti-donor T-cell sensitization before transplantation may help to identify patients at increased risk of acute cellular rejection, particularly in the early phases after kidney transplantation, and thus guide decision-making regarding the use of induction therapy.

## INTRODUCTION

Outstanding progress has been made in recent decades in assessing the humoral alloimmune sensitization against donor HLA antigens in kidney transplant patients, and has led to a major reduction in acute antibody-mediated rejection (ABMR) rates immediately after transplantation. However, no comparable success has been achieved in the monitoring of the anti-donor T-cell immune response. As a consequence, acute T-cell mediated rejection (TCMR) is still an unpredictable event, and this uncertainty negatively affects decision-making in daily clinical practice.

In fact, there is a considerable inconsistency between what we know from basic immune biology and what we have learnt from clinical transplantation. It is well accepted that T cells are key initiators, mediators and effectors of the alloimmune response, thus playing a key role in allograft rejection [1–3]. In fact, alloreactive memory/effector T cells are considered the hallmark of adaptive immunity since, compared to their naïve counterparts, they are long lived, can be fully reactivated with less co-stimulation, are less susceptible to novel immunosuppressants and are directly influenced by heterologous immunity [4–11]. Bearing this in mind, the impact of pre-transplant T-cell sensitization is more likely to take place during the initial period after transplantation, since preformed memory T cells are ready to cross-react to donor alloantigens, ultimately leading to allograft rejection.

Importantly, monitoring T-cell sensitization against donor or even a panel of reactive antigens has been shown to be feasible and reliable using the highly sensitive IFN-γ ELISPOT assay, and has also been shown to correlate with worse allograft function after kidney transplantation [12–17]. In this regard, our group recently reported the results of a non-randomized prospective clinical trial [18], monitoring anti-donor cellular alloreactivity in 60 kidney transplant recipients both before and six months after transplantation, with the aim of guiding immunosuppression for a calcineurin-inhibitor (CNI)-based or a CNI-free immunosuppressive regimen [18]. Interestingly, while very low rates of biopsy-proven acute rejection (BPAR) were obtained in both groups, even among T-cell sensitized individuals receiving CNI drugs a strong association was observed between 6-month persistence or *de novo* donor-specific T-cell alloreactivity and subclinical TCMR in protocol biopsies, suggesting a specific time-frame relationship between preformed donor-specific memory T cells or *de novo* alloreactive naïve T cells and their impact on kidney allograft outcome.

Here, we analyzed the presence of pre-transplant donor-specific T-cell sensitization in a large consecutive cohort of 90 kidney transplant recipients in whom the type of immunosuppression was given without knowing their baseline anti-donor T-cell sensitization status and the data obtained was further validated in a new independent group of kidney transplant recipients (n = 67). We aimed to investigate the main clinical variables associated with cellular sensitization and the specific post-transplant time-frame in which preformed donor-specific (d-s) memory T cells may negatively challenge allograft outcome.

## MATERIALS AND METHODS

### Patients

Ninety adult kidney transplant recipients from our Renal Transplant Unit at Bellvitge University Hospital (n = 90) attended between 2011 and 2012 were retrospectively analyzed on the basis of the availability of donor stimulator cells consisting of donor splenocytes in deceased-donor transplants or of donor peripheral blood mononuclear cells (PBMC) in living-donor transplants. The Bellvitge University Hospital ethics committee specifically approved the study, and all patients gave written informed consent. None of the transplant recipients had received immunosuppression before providing PBMCs to perform the ELISPOT assay. The pre-transplant ELISPOT result was not available to the clinicians before or after transplantation, and so had no influence on the choice of immunosuppression and clinical management after the transplantation. The mean time of follow-up of this cohort was 21 months (range 6–48 months).

Main baseline demographic variables in all patients were collected at the time of enrollment and included donor source and age, time on dialysis, number of human antigen leukocyte (HLA) mismatches, number of previous transplants, presence of donor-specific circulating antibodies by solid phase assays (Luminex®), cause of end-stage renal disease (ESRD) and recipient ethnicity (caucasian or otherwise). Furthermore, most relevant clinical variables associated with clinical transplant outcome such as type of maintenance IS (CNI-based or not), steroid withdrawal, use and type of induction therapy (T-cell depletion or not), delayed graft function (DGF), six and twelve-month allograft function (both the estimated glomerular filtration rate, eGFR, ml/min; and serum creatinine, μmol/L), CMV infection, biopsy-proven acute rejection (BPAR), type (antibody-mediated, ABMR; or T-cell mediated, TCMR) and time of rejection (early, <2 months or late, >2 months) were also pooled together for the analysis. DGF was defined as the need for dialysis during the first week after transplantation. The use of either T-cell depleting or monoclonal antibody induction therapy was based on the observed presumable risk derived from the percentage of PRA and HLA mismatch data. CNI were used as maintenance IS treatment in all but two patients who received mTor-inhibitors (Everolimus) as main immunosuppression.

Estimated glomerular filtration rate (eGFR) was calculated using the simplified Modification of Diet in Renal Disease equation (MDRD). All patients in the study showed a negative pre-transplant Complement-dependent cytotoxic (CDC) cross-match test.

A second independent validation cohort of kidney transplant patients (n = 67) was used to evaluate the predictive value of pre-transplant donor-specific T-cell alloreactivity on the advent of early T-cell mediated allograft rejection. These set of patients were consecutively transplanted from November 2011 to October 2013. The clinical management of all these patients was done irrespective of the pre-transplant donor-specific T-cell Elispot result, as the test was not done.

### HLA typing

Automated nucleotide sequencing was performed from genomic DNA by selective amplification (PCR) of target exons from each locus for a particular allele. Loci sequenced included HLA class I (A, B, and C) and II (DRB1/3/4/5 and DQA1/DQB1). Nucleotide sequencing was done as previously described [19].

### Alloantibody detection

Screening for circulating anti-HLA class I and II alloantibodies in peripheral blood was done by FlowPRA screening beads (One-lambda Inc.). Antibody specificities of positive samples were determined as previously described [20] using the LabScreen Single Antigen assay (One-lambda Inc.).

### IFN-γ ELISPOT assays and donor and recipient cell source

Donor-specific IFN-γ ELISPOT assays were done following recently described techniques [18,21]. Peripheral blood samples were obtained in heparinized tubes from renal transplant recipients before kidney transplantation. Donor cells were obtained from donor spleens or peripheral blood mononuclear cells (PBMCs) in deceased and living donors respectively. PBMCs and splenocytes were isolated by standard Ficoll density gradient centrifugation and were frozen in liquid nitrogen and subsequently used for the IFN-γ ELISPOT assay. Deceased-donor splenocytes were CD2-depleted (Easysep® Human CD2 Selection kit, StemCell, France) and living-donor PBMCs were CD3-depleted (human CD3+ Cell Depletion Cocktail, RosetteSep® kit, StemCell, France) and tested in triplicate wells with respective recipient PBMCs. A positive d-s ELISPOT test was considered as ≥25 IFN-γ spots/3x10^5^ PBMC.

### Renal allograft histology

Renal allograft biopsies were performed in patients undergoing acute clinical graft dysfunction. All renal histologies were analyzed following the Banff ‘09 score, and histological analyses were blindly evaluated.

### Statistical analysis

All data are presented as mean ± sd. Groups were compared using the X^2^-test for categorical variables and the one-way analysis of variance or Student’s t-test for normally distributed data for quantitative variables, and the nonparametric Kruskal-Wallis or Mann-Whitney U-test for non-normally distributed variables.

A sensitivity/specificity ROC curve analysis was done to determine the ELISPOT test value for the prediction of the advent of early T-cell mediated acute rejection. Stepwise linear regression and binary logistic regression analysis were performed to determine the independent correlation of several independent variables with the presence of early cellular acute rejection. The statistical significance level was defined as p<0.05.

## RESULTS

### 1. Main clinical and demographic variables associated with anti-donor T-cell sensitization

**Fig 1:**
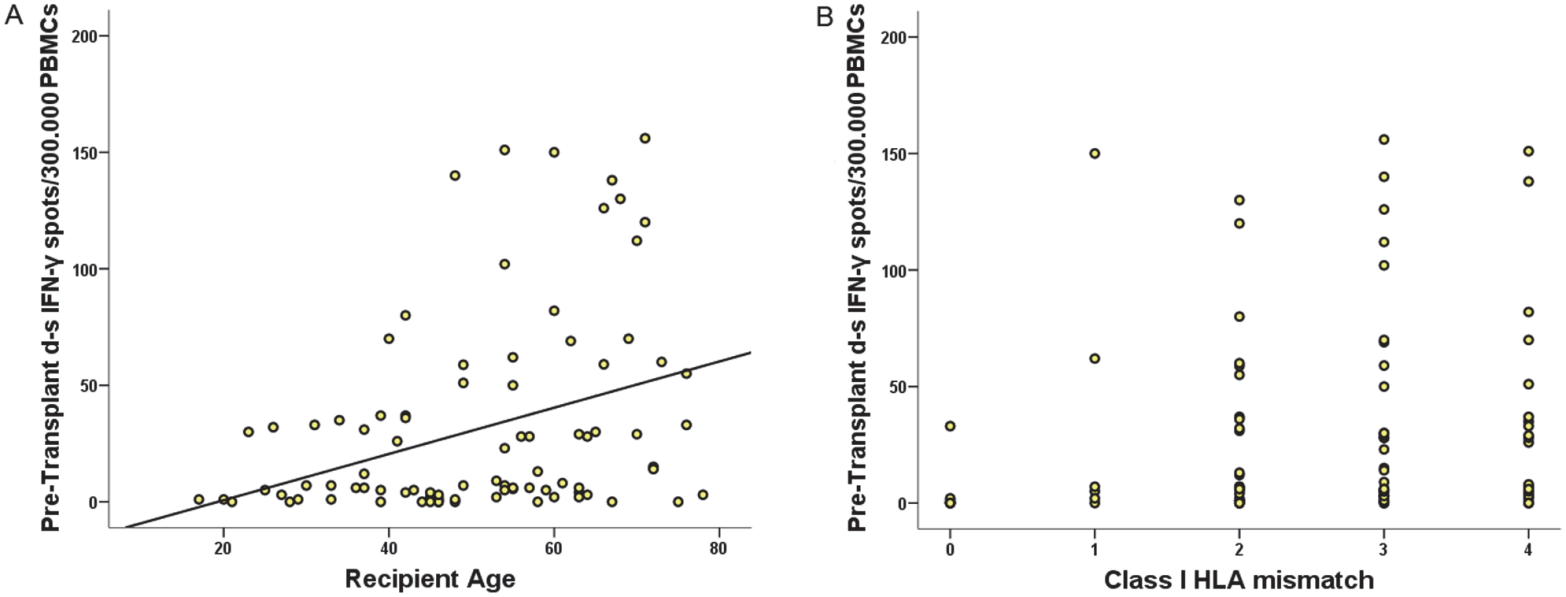
Pre-transplant d-s T-cell alloreactivity positively correlates with recipient age and with HLA class I mismatch. A. Correlation between pre-transplant frequency of donor-specific IFN-γ T-cell spots and recipient age (years) (R = 0.349, p = 0.001). B. Correlation between pre-transplant frequency of donor-specific IFN-γ T-cell spots and class I HLA mismatches (R = 0.207, p = 0.05).

The main baseline clinical and demographic variables were assessed among all patients for their association with pre-transplant T-cell sensitization. As shown in Table 1, 37/90 (41.1%) had detectable anti-donor alloreactivity pre-transplantation whereas 53/90 (58.9%) did not. No differences were found regarding the number of previous transplants, cause of ESRD, ethnicity or time on dialysis. No association was observed with the degree of pre-transplant humoral sensitization of either donor or non-donor-specific anti-class I and II HLA antibodies. Interestingly, anti-donor T-cell alloreactive patients tended to be older individuals receiving allografts from older deceased donors with low HLA class I matching (Fig. 1). Regarding the validation cohort, there were more T-cell sensitized patients than non T-cell sensitized, but were all of them comparable regarding main clinical and immunological characteristics.

**Table 1:** Patients’ demographic characteristics.

	Training Set (n = 90)			Validation Set (n = 67)		
Baseline demographics	Positive PRE-TX d-s ELISPOT (n = 37; 41%)	Negative PRE-TX d-s ELISPOT (n = 53; 59%)	P value	Positive PRE-TX d-s ELISPOT (n = 46; 68.7%)	Negative PRE-TXd-s ELISPOT (n = 21; 31,3%)	P value
Donor age (years)	60.3±16	51±12.26	0.002	57.4±16.9	50.4±10.8	0.05
Recipient age (years)	55±14.6	48±14.20	0.02	52±15	54,1±9.7	0.50
Recipient gender (F) (%)	15 (40.5)	15 (28.3)	0.26	12 (26.1%)	7 (33.3)	0.77
Caucasian ethnicity (%)	33 (89.2)	51 (9796.2)	0.22	43 (95,6%)	20 (95,2%)	0,78
Cause of ESRD			0.25			0,36
- Unknown (%)	11 (29.7)	13 (24.5)		16 (35.6)	4 (18,2)	
- Glomerular (%)	9 (35.9)	19 (32.4)		11 (24,4)	6 (27,3)	
- Intersticial (%)	7 (18.9)	13 (24.5)		7 (15.2)	2 (9.5)	
- Vascular (%)	4 (10.8)	2 (3.8)		5 (11,1)	6 (27,3)	
- Diabetes (%)	4 (10.8)	6 (11.3)		2 (4.3)	0 (0)	
Donor type (Living) (%)	10 (27)	40 (75.5)	<0.001	9 (20,5%)	7 (31.8%)	0,36
Number of previous transplants (<1TR/>1TR) (%)	7(18.9) / 30(81.1)	6(11.3) / 47(88.7)	0.31	5(11) / 40(87)	2(9.5) / 19(90.5)	0.85
Dialysis duration (month)	45.2±45	42.3±46.7	0.76	34.4±19.7	36.1±30.6	0.83
Number Class I HLA mismatch	2.83±1	2.4±1.15	0.06	2.89±0.68	2.19±1.37	0.04
Number Class II HLA mismatch	1.16±0.5	1.16±0.76	0.95	1±0.57	0.95±0.67	0.76
HLA Class I Ab (Pos) (%)	4 (10.8)	5 (9.5)	0.9	2(4.3%)	3 (14.3%)	0,15
HLA ClassII Ab (Pos) (%)	6 (16.2)	9 (17)	0.9	5(10.9%)	2 (9.5%)	0,87
DSA (Pos) (%)	2 (5.4)	4 (7.5)	0.9	0 (0)	3 (13,6)	0.01
- HLA ClassI DSA (%)	0(0)	1 (11.1)	0.4	0 (0)	2 (66.6)	0,03
- HLA ClassII DSA (%)	2 (33.3)	4 (44.4)	0.7	0 (0)	1 (33.3)	0,14
rATG/Bxmab/No induction (%)	12(32.5) /22(59,5) / 3(8)	14(26) / 29(55) / 10(19)	0.35	4(8,7) / 38(82,6) / 4(8,7)	6(28,6) / 12(57,1) / 3(14,3)	0.07
CNI-IS (TAC) (%)	33 (89.2)	43 (84.3)	0.51	44 (95.7)	19 (90.5)	0.41
Steroid withdrawal (<4 weeks) (%)	4 (10.8)	13 (24.5)	0.10	18 (39.1)	12 (57.1)	0.19
d-s IFN-γ spots/3x10^5^PBMC	69.34±42.7	4.7±5.6	<0.001	97±65,4	12.4±15.6	<0.001

Variables associated with pre-transplant donor-specific T-cell allosensitization.

APKD, autosomic polycystic kidney disease; D, deceased; d-s, donor-specific; DSA, donor-specific antigen; ESRD, end stage renal disease; F, female; HLA, human leukocyte antigen; L, living; M, male; Neg, negative; PBMCs, peripheral blood mononuclear cells; PreTX, pre-transplant; Ab, antibodies; PRA, panel reactive antibodies; Pos, positive; TR, transplants.

Two patients died during the first year; neither presented pre-formed donor-specific T-cell sensitization. One died of a cardiac arrest nine months after transplantation and the other of an opportunistic infection caused by *pneumocystis juvencii*. Three allografts were lost within the first year, one due to a vein thrombosis, another due to unresolved urine leakage (both were non-sensitized cellular transplant individuals), and a third in a highly T and B-cell sensitized patient due to a severe BPAR (mixed ABMR and TCMR) who did not respond to intensive rescue immunosuppressive treatment.

### 2. Preformed anti-donor T-cell alloreactivity is associated with early acute TCMR and worse allograft function after kidney transplantation

**Fig 2:**
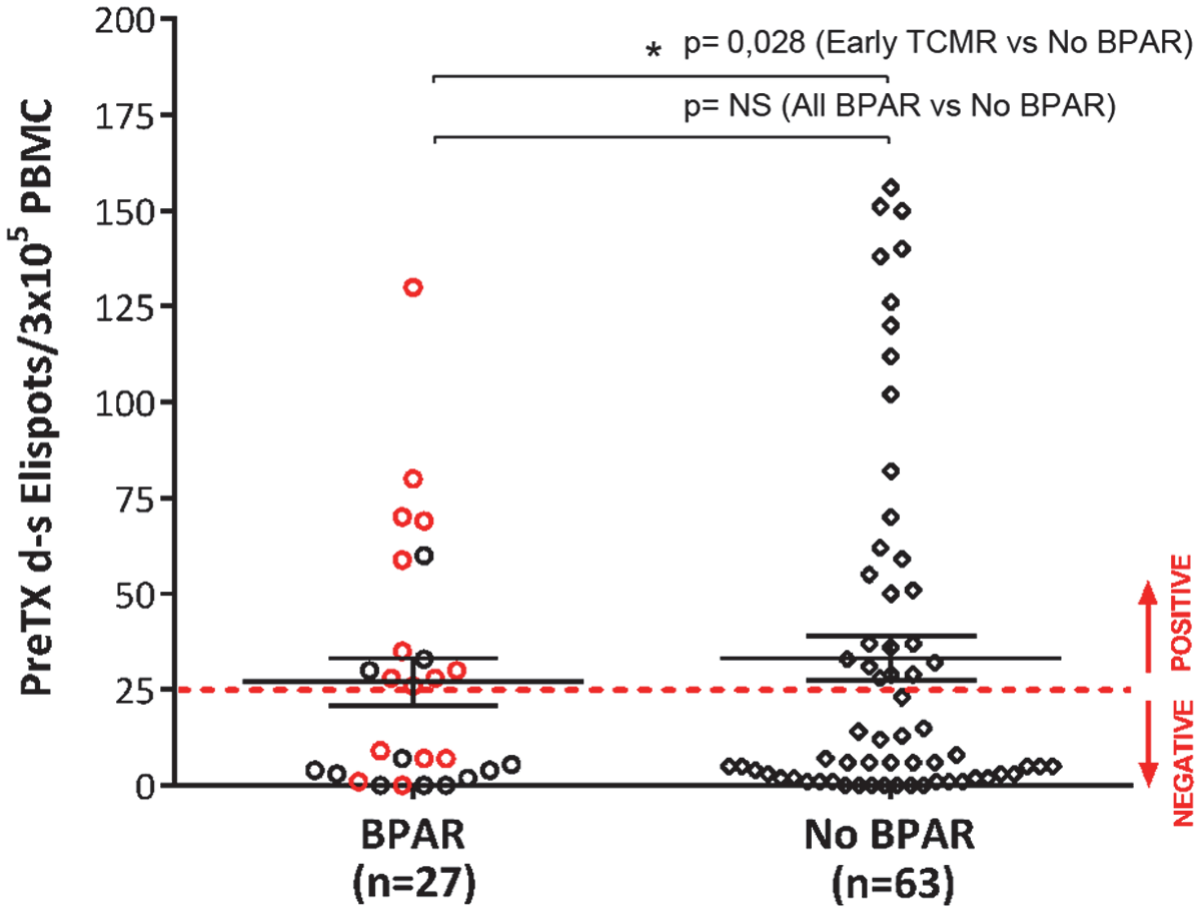
Pre-transplant d-s T-cell alloreactivity and early TCMR. No differences between preformed donor-specific T-cell alloreactive responses and incidence of all types of BPAR were observed. Nevertheless, kidney transplant patients developing early TCMR (< 2 months after transplantation) showed significantly higher preformed donor-specific T-cell sensitization than those that did not (10/37;27% vs 5/53;9.4%, p = 0.028). Red dots represent pre-transplant T-cell alloreactive kidney transplant patients developing early TCMR.

T-cell alloreactive and non-alloreactive patients were comparable in terms of main baseline clinical variables such as the use and type of induction therapy, the choice of maintenance immunosuppression (principally based on CNI drugs, either Cyclosporine-A or Tacrolimus) as well as regarding steroid withdrawal (Table 2). High pre-transplant anti-donor alloreactive patients showed higher incidence of DGF but similar incidence of CMV infection. When the global incidence of BPAR was analyzed, no differences were found between T-cell alloreactive and non-alloreactive recipients (Fig. 2); neither ABMR nor TCMR were associated with pre-formed donor-specific T-cell alloreactivity. Similarly, no association was observed between pre-transplant T-cell ELISPOT and either graft loss or patient death.

**Table 2:** Impact of pre-transplant d-s T-cell allosensitization on allograft outcome.

Main clinical events (N = 90)	Positive PreTX d-s T-cell ELISPOT (n = 37; 41%)	Negative PreTX d-s T-cell ELISPOT (n = 53; 59%)	p-value
DGF (yes/no) (%)	16 (43.2) / 21 (56.8)	12 (22.6) / 41 (77.4)	0.03
CMV infection (yes/no) (%)	3 (8.1) / 34 (91.9)	8 (15.19) / 45 (84.9)	0.31
BPAR (yes/no) (%)	13 (35.1) / 24 (64.9)	14 (26.4) / 39 (73.6)	0.37
- Type (TCMR, ABMR) (%)	11 (84.6) / 2 (15.4)	11 (78.5) / 3 (21.5)	0.56
- Time BPAR (<2mo/>2mo) (%)	12 (92.3) / 1 (7.7)	6 (57.1) / 8 (42.9)	0.01
- Early TCMR (yes/no) (%)	10 (27) / 27 (73)	5 (9.4) / 48 (90.6)	0.02
Graft loss (yes/no) (%)	1 (2.7) / 36 (97.3)	4 (7.6) / 49 (92.5)	0.32
Exitus (yes/no) (%)	0 (0) / 37 (100)	2 (3,8) / 51 (96,2)	0,23

PreTX: pre-transplant; BPAR, biopsy-proven acute rejection; CMV, cytomegalovirus; DGF, delayed graft function; TCMR, T-cell mediated rejection; ABMR, antibody-mediated rejection.

**Fig 3:**
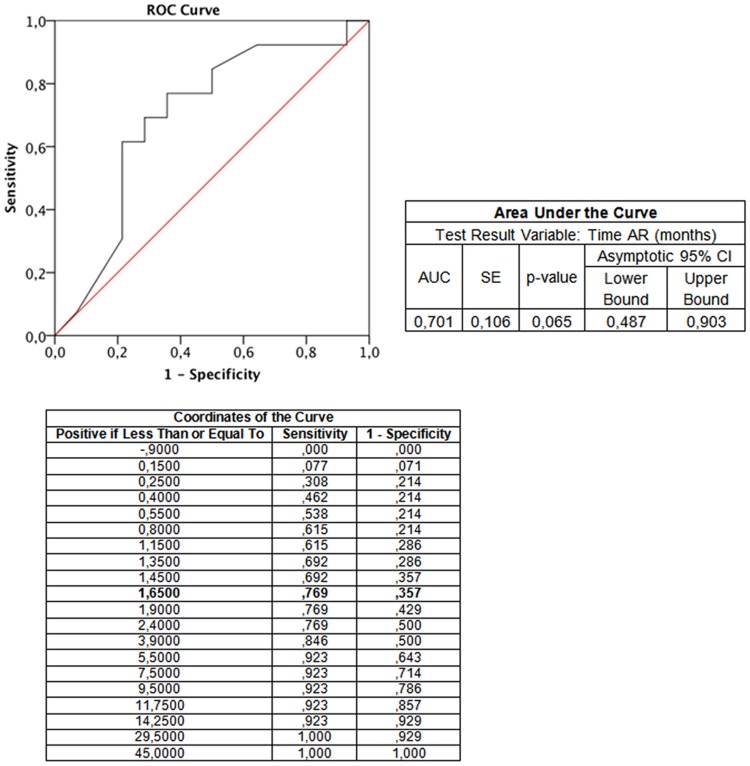
Receiver operating characteristic (ROC) curve estimating the most likely time-frame of TCMR associated with pre-transplant T-cell sensitization. Positive pre-transplant T-cell sensitization was a highly sensitive and specific predictor of the advent of TCMR during the first 8 weeks (1.65 months) after transplantation (AUC = 0.701; p = 0.065).

In order to establish whether any specific time frame could most likely distinguish all TCMR events significantly associated with pre-transplant T-cell sensitization, a receiver-operating characteristic (ROC) curve analysis was carried out taking into account all time-points of BPAR episodes and the pre-transplant ELISPOT data. Interestingly, the first eight weeks after transplant proved to be the most accurate time frame for identifying TCMR episodes that were related to the pre-transplant donor-specific T-cell immune response, with a notably high sensitivity and specificity (sensitivity = 75%, specificity = 80%) (AUC = 0.701, p = 0.065) (Fig. 3). Now, using this time-period as a binary variable, a significant association was obtained between the pre-transplant anti-donor T-cell sensitization and the occurrence of early TCMR, but it did not influence the occurrence of other rejection events (p = 0.022) (Fig. 2).

Even though pre-transplant anti-donor alloreactive patients showed worse 6 and 12-month allograft function, these values were probably influenced by the significant higher donor age of alloreactive transplant recipients as compared to non-alloreactive recipients (data not shown). Nonetheless, a numerically worse allograft function was consistently observed among pre-transplant alloreactive patients when stratified both by type of kidney transplant (living or deceased donors) as well as by donor age (older or younger than 50 years old) (data not shown).

### 3. Young, highly pre-transplant T-cell alloreactive recipients show particularly high risk of early TCMR after kidney transplantation

**Fig 4:**
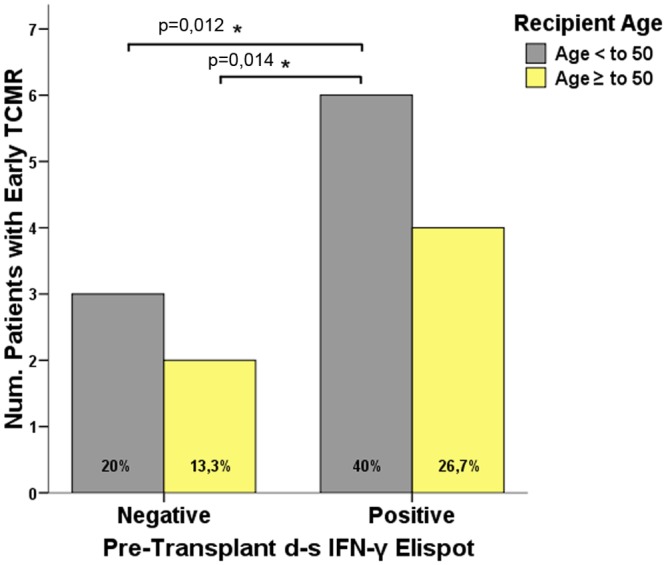
Incidence of early TCMR is higher within young T-cell alloreactive transplant recipients than in elderly patients. Pre-transplant T-cell alloreactive individuals younger than 50 years old (R<50/ELISPOT+) showed significantly higher incidence of early TCMR than young patients with a negative T-cell ELISPOT (R<50/ELISPOT-) and elderly patients with either a positive (R>50/ELISPOT+) or negative pre-transplant ELISPOT (R>50/ELISPOT-), (6/15;40%, 3/15;20%, 4/15;26.7%, 2/15;13.3%, respectively, p = 0.030). Statistically significant differences were found between alloreactive individuals below 50 (R<50/ELISPOT+) and non-alloreactive patients pre-transplantation [(R<50/ELISPOT-) and (R>50/ELISPOT-)] (p = 0.012 and p = 0.014 respectively). A trend toward a significant difference was observed between patients under 50 and elderly alloreactive patients (p = 0.091).

Since pre-transplant T-cell sensitization correlated positively with both recipient and donor ages and are matched for transplant selection, we were particularly interested in determining whether recipient age and pre-transplant cellular sensitization could influence the risk of early TCMR. Therefore, we stratified recipient age as a binary variable (below or above 50 years), and evaluated the risk of early TCMR depending on the pre-transplant ELISPOT test (R<50/ELSPOT- (n = 30); R<50/ELSPOT+ (n = 14); R>50/ELSPOT- (n = 23) and R>50/ELSPOT+ (n = 23). As shown in Fig. 4, a significantly higher incidence of early TCMR was observed among young highly alloreactive individuals as compared to all other groups (R<50/ELSPOT- (3/15, 20%); R<50/ELSPOT+ (6/15, 40%); R>50/ELSPOT- (2/15, 13.3%) and R>50/ELSPOT+ (4/15, 26.7%); p = 0.03). This data was further confirmed when we put all patients of the discovery and prediction set together (n = 157) (R<50/ELSPOT- (3/24, 12.5%); R<50/ELSPOT+ (10/24, 41%); R>50/ELSPOT- (3/24, 12.5%) and R>50/ELSPOT+ (8/24, 33%); p = 0.05).

### 4. T-cell depletion provides protection against early TCMR in highly donor-specific T-cell alloreactive patients

**Fig 5:**
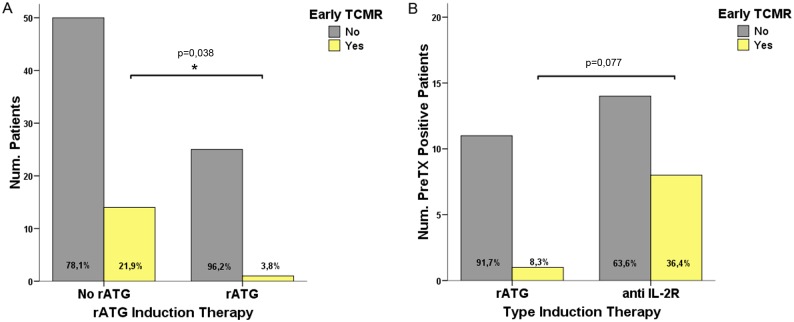
T-cell depletion induction therapy provides protection against early TCMR. **5a**. Patients receiving T-cell depletion as induction therapy showed significantly lower incidence of early TCMR [3.8% (1/26) vs 22% (14/64) p = 0.038] than those that did not.**5b**. Incidence of early TCMR in anti-donor T-cell sensitized patients receiving either anti-IL2 receptor blockade or rATG. A higher incidence of early TCMR was observed among highly T-cell sensitized patients receiving anti-IL2 receptor blockade than in patients receiving rATG [36.4% (8/22) and 8% (1/12); p = 0.07 respectively].

Whether the use and type of induction therapy could impact on preformed highly alloreactive anti-donor T-cell frequency was further evaluated. While the use of any type of induction therapy did not discriminate those patients developing any kind of BPAR (data not shown, p>0.05), patients receiving T-cell depletion with rATG showed a significantly lower incidence of early TCMR as compared to those that did not (1/26; 3,8% vs 14/64; 22%, respectively, p = 0.038) (Fig. 5a). Furthermore, when only highly donor-specific alloreactive patients were analyzed in relation to their type of induction therapy, eight out of 22 (36.4%) patients receiving anti-IL2R (basiliximab) developed early TCMR, compared with only one out of 12 (8.3%) patients receiving rATG (0.07) (Fig. 5b). Of note, no protective effect by rATG was observed when non T-cell alloreactive transplant patients were evaluated (4/38; 10.5%, non-sensitized patients not receiving rATG experienced early TCMR vs 1/15, 6% developed early TCMR, p = NS).

### 5. Absence of Pre-transplant d-s T-cell alloreactivity as a significant protective variable for developing early TCMR

The most relevant clinical variables influencing the outcome of early TCMR (ethnicity, cold ischemia time, use of T-cell depletion, number of HLA mismatches, the advent of DGF, type of donor as well as pre-transplant T-cell sensitization) were assessed with univariate and multivariate Cox regression analysis. Although non-caucasian ethnicity, longer ischemia time, non-use of T-cell depletion and a positive pre-transplant ELISPOT were associated with an increased risk of early TCMR, only the presence of baseline anti-donor T-cell sensitization and non-use of T-cell depletion as induction therapy were significant independent correlates of risk for early TCMR (Table 3).

**Table 3:** Univariate and step-wise multivariate analyses of variables predicting early T-cell mediated rejection (TCMR).

Variables predicting Early TCMR (N = 90)	Univariate analysis			Multivariate analysis		
	RR	CI 95%	p	RR	CI 95%	p
Caucasic ethnicity	6	1.082–33.274	0.04	0.2	0.021–1.960	0.16
Cold ischemia time (hours)	1.06	1.001–1.139	0.04	1.04	0.971–1.129	0.23
PreTX d-s IFN-γ ELISPOT (negative)	0,28	0.087–0.908	0.03	0.23	0.061–0.891	0.03
rATG induction	0,14	0.018–1.001	0.05	7.45	0.819–67.74	0.07
HLA mismatch (≤3)	0,31	0.083–1.221	0.09	0.18	0.028–1.227	0.08
DGF (yes)	0,44	0.143–1.380	0.16	2.38	0.34–16.74	0.38
Donor (Living)	3.00	0.932–9.653	0.06	0.01	0.001–2.30	0.1

CI, confidence interval; DGF, delayed graft function; rATG, rat anti-thymocyte globulin;

RR, relative risk.

### 6. Verification of pre-transplant anti-donor T-cell sensitization as a risk factor for TCMR in a new cohort of kidney transplant patients

The impact of preformed donor-specific T-cell alloreactivity was validated in a second independent cohort of kidney transplant patients with similar baseline clinical and immunological characteristics. As shown in Table 4, although a rather low specificity and positive predictive value was obtained, a high sensitivity and negative predictive value (88.9% and 95.2%, respectively) was observed for the prediction of early TCMR (Table 4). Similarly to the training set, highly alloreactive patients receiving T-cell depletion as induction therapy showed lower incidence of TCMR as compared to those receiving either basiliximab or no induction therapy (0/4 (0%) vs 12/38 (31,6%) vs 1/4 (25%), p = 0.06, respectively).

**Table 4:** Predictive value of IFN-γ ELISPOT assay for early TCMR in an independent validation cohort.

	Predictive value			
Variable	Sensitivity	Specificity	NPV	PPV
PreTX d-s IFN-γ ELISPOT (≥25 IFN-γ spots /3×10^5^ PBMC)	88.9%	62.5%	95.2%	40%

NPV, Negative predictive value; PreTX, pre-transplant; Pos, Positive; Neg, Negative.

## DISCUSSION

In this study, we show that high frequencies of donor-specific alloreactive memory/effector T cell responses before kidney transplantation is frequent among patients waiting for a kidney allograft. Furthermore, we report for the first time that this preformed anti-donor T-cell sensitization seems to have a direct negative impact on kidney allograft outcome by favoring TCMR during the early period of time after transplantation, especially among highly T-cell sensitized individuals against donor antigens and patients not receiving T-cell induction therapy. Of note, this observation was further validated in a subsequent independent cohort of kidney transplant patients with high sensitivity and negative predictive value.

Unlike acute antibody-mediated rejection (ABMR), TCMR remains an unpredictable process in clinical practice since no accurate immune monitoring is currently done. However, in recent years, a strong deleterious association has been observed between the presence of alloreactive memory T cells and clinical and also subclinical immune-mediated allograft injury or allograft dysfunction in humans [12,13,22,23]. In this study, using the highly sensitive IFN-γ ELISPOT assay, we emphasize that these immune-mediated events driven by preformed donor-specific memory T-cell clones may not be predictable on the basis of epidemiological backgrounds and may occur during the first weeks after transplantation. This is significant since it suggests that delayed clinical or subclinical TCMR might be driven by either persistent or rather, by *de novo* naïve T-cell activation, potentially reflecting insufficient immunosuppressive exposure. In fact, in the multivariate analysis performed, the presence of high frequencies of circulating donor-specific memory/effector T cells prior to transplant surgery was shown to be an independent predictor of early TCMR, but not of all the immune-mediated clinical events occurring later on. A plausible explanation of the relatively frequent detection of anti-HLA cellular alloreactivity before kidney transplantation may be heterologous immunity, in which T cells, initially primed against infectious agents and environmental antigens, cross-react with allogeneic MHC molecules [24,25]. Nevertheless, whether this or other mechanisms are responsible for the presence of alloreactive memory T-cell responses in the absence of clear allogeneic sensitization deserves further investigation.

A conclusion that can be drawn from our study is the importance of an optimal HLA matching, especially within class I molecules between donor/recipient pairs in order to reduce the chance that pre-formed memory T cells recognize donor alloantigens. Likewise, we recently showed that the higher the number of HLA mismatches, the greater the frequency of donor-specific memory T-cell responses, particularly among T-cell subsets primed by the direct pathway of alloantigen presentation [26]. Even though not tested in our study, this finding and those reported by others [12–17] suggest a role for both CD8+ and CD4+ memory T cells in the anti-donor allogeneic immune response. Furthermore, as previously shown [18,26,27], the pre-transplant anti-donor humoral allosensitization did not illustrate the allospecific cellular immune response, emphasizing the importance of monitoring both effector mechanisms of adaptive immunity before transplantation.

Even though older individuals showed increased frequencies of alloantigen-specific memory T cells as compared to younger patients, younger T-cell alloreactive individuals seem to have a more effective anti-donor effector immune response, as shown by the significantly higher incidence of early TCMR particularly among younger alloreactive T-cell sensitized patients. A plausible explanation is that although aged recipients exhibit higher numbers of memory T cells with a broader antigen *repertoire*, these cells have a significantly poorer capacity to mount effective recall effector responses compared with young memory/effector T cells [28–30]. Interestingly, Hricik and colleagues [31] recently reported the increased risk of TCMR and poorer graft function in patients receiving kidney allografts from older deceased donors, suggesting that the more inflammatory *milieu* triggered in these grafts could facilitate a much more effective effector T-cell response among T-cell sensitized individuals due to the higher immunogenicity. In our study, although a similar higher T-cell sensitization was found among individuals receiving older allografts, the most relevant variable influencing the outcome was recipient’s age. This observation stresses the fact that transplant rejection is much more likely to happen in highly T-cell sensitized younger individuals, whereas in older sensitized transplant patients it would be facilitated if receiving highly immunogenic tissues from older donors.

An important issue still under debate in clinical kidney transplantation is the choice and type of induction therapy. In our study we found that the use of T-cell depletion as induction therapy with rATG, in combination with CNI drugs, seemed to provide significantly better protection for T-cell sensitized patients against developing TCMR than the use of anti-IL2 receptor monoclonal antibodies or no induction therapy. Indeed, we found a significant reduction of early TCMR among highly T-cell sensitized patients receiving rATG as compared to transplant recipients using other type of induction treatment or no induction, whereas the use of rATG did not provide any benefit as compared to other induction treatments when given in non T-cell alloreactive individuals thus, suggesting the usefulness of this assay to individualize the use of such aggressive induction therapy. This finding is in fact in line with previous reports from others and us [18,32,33] advocating the use of induction therapy, particularly T-cell depletion, to prevent post-transplant T-cell mediated rejection among highly T-cell sensitized individuals.

The new observations reported in this work underline the high interest of this assay for being implemented in clinical practice as it provides crucial information to transplant clinicians not currently available; on the one hand, the likelihood of T-cell mediated rejection in the very early phases after transplantation that may not be inferred with patients’ baseline clinical background on the other, help further refine decision-making regarding the use of T-cell depletion as induction therapy, particularly among the most fragile transplant population, that is in the older range of age, in whom in absence of humoral sensitization, this potent immunosuppression could be safely avoided.

A main limitation of this study is its retrospective nature. However, the multivariate analysis performed, together with the results obtained, which corroborate those of some previous reports, strengthen our observations and should alert the transplant community to the urgent need to perform prospective, observational studies as well as interventional trials to test our results.

The high sensitivitiy and particularly high negative predictive value of the ELISPOT test for early TCMR is of great relevance due to its capacity to rule out the disease. Therefore, patients with no evidence of T-cell sensitization would be suitable candidates to enroll in such clinical trials, which should ideally be conducted under the auspices of international collaborative networks.

## CONCLUSIONS

High levels of donor-specific alloreactive memory T cells may be relatively frequent prior to transplantation, despite the absence of any clinically recognizable sensitizing events. They may directly facilitate the advent of TCMR early after transplantation, particularly in patients not receiving T-cell depleting agents as induction therapy. Therefore, screening for anti-donor T-cell sensitization should be seriously considered in kidney transplant patients. At this point, prospective randomized trials are clearly warranted.
